# Expanding horizons: radiolabeled FAPI in imaging of autoimmune diseases

**DOI:** 10.3389/fimmu.2026.1797336

**Published:** 2026-03-04

**Authors:** Jiaoniu Duan, Jinfang Gao, Gailian Zhang, Liyun Zhang

**Affiliations:** 1 Third Hospital of Shanxi Medical University, Shanxi Bethune Hospital, Shanxi Academy of Medical Sciences, Tongji Shanxi Hospital, Taiyuan, China; 2 Department of Rheumatology and Immunology, Fifth Hospital of Shanxi Medical University, Shanxi Provincial People's Hospital, Taiyuan, China

**Keywords:** AIDs, autoimmune diseases, FAP, FAPI, fibroblast activation protein

## Abstract

Fibroblast activation protein alpha (FAP) is a type II transmembrane protease that exhibits both dipeptidyl peptidase and endopeptidase activities. It is upregulated in activated fibroblasts, particularly in tumor stroma and in fibrotic processes associated with various benign conditions. Recent studies have revealed that FAP is also highly expressed in certain Autoimmune Diseases (AIDs) characterized by chronic inflammation and tissue remodeling. Small-molecule inhibitors targeting FAP (FAPI) positron emission tomography (PET) provides a direct imaging modality for assessing fibroblast activation *in vivo*. FAPI’s application in oncology has attracted considerable attention in nuclear medicine. However, evidence supporting its efficacy in AIDs remains limited. This review systematically evaluates the diagnostic and phenotyping potential of FAPI PET in AIDs, expands its applications across a broader disease spectrum, highlights technical advantages, and delineates future research directions.

## 1 Introduction

AIDs represent a diverse group of conditions characterized by immune dysregulation, which leads to persistent inflammation and tissue damage (1). Epidemiological studies reveal that AIDs affect a significant portion of the global population, with prevalence rates in high-income countries ranging from 3% to 8% (2). This category of diseases includes rheumatoid arthritis, inflammatory bowel disease, and Spondyloarthritis, each contributing substantially to global morbidity and mortality, resulting in significant social and economic burdens (1). Consequently, understanding the mechanisms are critical for effective diagnosis, treatment, and management.

Under physiological conditions, fibroblasts play a key role in the continuous remodeling of connective tissue by synthesizing extracellular matrix components such as collagens and proteoglycans. Fibroblasts are resident in multiple organs, including the heart, lungs, skeletal muscle, and skin (3). In the inflammatory microenvironment of AIDs, fibroblasts are markedly activated, leading to fibrosis and subsequent organ damage (3). The interaction between activated fibroblasts and adjacent immune cells significantly impacts AIDs progression. Activated fibroblasts are known to produce inflammatory cytokines and chemokines that sustain chronic tissue inflammation and remodeling associated with these diseases (3).

FAP, a type II transmembrane protease with dipeptidyl peptidase and endopeptidase activities, is upregulated in activated fibroblasts (4). In 2018, a significant advancement in the field of nuclear medicine research occurred particularly concerning tumors and fibrotic diseases when Heidelberg University researchers introduced the first quinolone-based FAPI (5). FAPI are recognized for their high affinity and specificity, offering innovative approaches for assessing fibroblast activation *in vivo* (4, 5). While FAPI PET’s oncological applications have been extensively characterized, there remains a paucity of comprehensive syntheses systematically evaluating its expanding utility specifically in AIDs. We summarize clinical studies on the role of FAPI PET in patients with AIDs up to December 2025. This review aims to provide an up-to-date and structured overview of the current evidence for FAPI PET in AIDs, with special emphasis on its diagnostic precision and its emerging role in treatment monitoring and risk stratification.

## 2 Methods

Search strategy: This narrative review synthesizes the current clinical evidence on the application of FAPI PET/CT in AIDs. To identify relevant studies, a systematic literature search was conducted in PubMed and Embase databases from inception to December 31, 2025. The search strategy combined the following key terms and their variants: (“fibroblast activation protein inhibitor” OR “FAPI”) AND (“PET” OR “positron emission tomography”) AND (“autoimmune” OR “rheumatoid arthritis” OR “spondyloarthritis” OR “Psoriatic Arthritis” OR “SAPHO syndrome” OR “IgG4-related disease” OR “systemic sclerosis” OR “myositis” OR “systemic lupus erythematosus” OR “lupus nephritis” OR “vasculitis” OR “Crohn’s disease”).

Inclusion criteria were: (1) original clinical studies (prospective or retrospective cohorts, case series, case report) involving human subjects; (2) investigation of FAPI PET/CT in non-oncologic, immune-mediated inflammatory or fibrotic conditions; (3) reporting of qualitative or quantitative imaging outcomes. Exclusion criteria included: (1) preclinical or animal studies; (2) review articles, editorials, commentaries, or conference abstracts without original data; (3) studies focused exclusively on malignant diseases.

Study selection process: The independent screening by two authors and resolution of disagreements by consensus.

Duplicate and overlap handling: Duplicate records identified across databases were removed prior to screening. For studies with overlapping patient cohorts, we prioritized the most comprehensive or recent publication to avoid data duplication.

Quality assessment approach: We clarify that given the narrative and rapidly evolving nature of the field, no formal risk-of-bias tool (e.g., QUADAS-2) was applied, and a meta-analysis was not undertaken. Our focus was instead on transparently reporting study characteristics and limitations.

To ensure transparency and comprehensiveness, we explicitly report for each included study its design, sample size, specific FAPI tracer used, comparator imaging modalities, key findings, and major methodological limitations. This approach aims to provide a systematic and balanced overview of the current evidence. A summary of the key studies is presented in **Table 1**.

**Table 1:** Summary of key clinical studies on FAPI PET/CT in AIDs.

Disease	Study design	Tracer(s) used	Sample size (n)	Key comparator(s)	Main findings	Major limitations
RA (8, 9, 11)	Prospective cohort study	[^68^Ga]Ga-FAPI-04, [^18^F]FAPI-42	18–42	[^18^F]FDG PET/CT, X-ray, Clinical scores (DAS28)	High detection sensitivity; FAPI parameters correlate with clinical disease activity.	Single-center; heterogeneous tracers; lack of histologic validation; small sample in sub-studies.
PsA (15)	Prospective cohort study	[^68^Ga]Ga-FAPI-04	16–36	Ultrasound, [^18^F]NaF PET/CT	Identifies subclinical enthesitis/synovitis; correlates with symptom counts.	Small sample size; limited longitudinal data for progression validation.
Axial SpA/AS (18–20)	Prospective cohort study, Case report	[^18^F]AlF-NOTA-FAPI-04, [^68^Ga]Ga-FAPI-46	20–22	MRI, [^18^F]FDG PET/CT	High sensitivity for sacroiliitis/spondylitis; correlates with ASDAS & CRP.	Single-center; different tracers limit comparability; no control for degenerative changes.
SAPHO Syndrome (23, 24)	Prospective comparative study	[^68^Ga]Ga-DOTA-FAPI-04	1–21	^99m^Tc-MDP bone scan, [^18^F]FDG PET/CT	Higher lesion detection rate vs. MDP & FDG; good symptom correlation.	Mostly small case series; heterogeneous disease activity; lack of standardized metrics.
IgG4-RD (28–30)	Prospective comparative study, Retrospective analysis	[^68^Ga]Ga-FAPI-04, [^18^F]AlF-NOTA-FAPI-04	30–50	[^18^F]FDG PET/CT	Distinguishes fibrotic vs. proliferative subtypes; PET index prognostic for relapse.	Retrospective components; tracer heterogeneity; need for histologic correlation in all lesions.
SSc (33–35)	Prospective cohort study, Proof-of-concept study	[^68^Ga]Ga-FAPI-04, [^18^F]AlF-NOTA-FAPI-04	14–31	HRCT, PFT, Cardiac MRI	Quantifies multi-organ fibrotic burden; baseline lung uptake predicts ILD progression.	Single-center pilot studies; physiological muscle/heart uptake may confound; no test-retest data.
IIM (38, 39, 41, 42)	Prospective cohort study, Case report	[^68^Ga]Ga-FAPI-04	14–21	[^18^F]FDG PET/CT	Assesses myositis & ILD activity; uptake correlates with CK/CRP and predicts ILD progression.	Small sample; inclusion of mixed phenotypes; variable treatment statuses.
LN (44, 45)	Prospective cohort study	[^68^Ga]Ga-FAPI-04, [^18^F]F-FAPI	20–29	Renal biopsy, [^18^F]FDG PET/CT	Uptake correlates with biopsy chronicity index; may predict treatment response.	Single-center; renal excretion affects background; need for validation in larger cohorts.
LVV (47–49)	Retrospective study, Case series	[^18^F]FAPI-42, [^68^Ga]Ga-FAPI-04	6–30	[^18^F]FDG PET/CT, MRI	High lesion contrast; detects remodeling phase activity in remission.	Small retrospective cohorts; mixed vasculitis types; tracer differences.
CD (53, 54, 56)	Prospective cohort study	[^68^Ga]Ga-FAPI-04	20–30	CT/MR enterography, [^18^F]FDG PET/CT, Endoscopy	Distinguishes fibrotic from inflammatory strictures	Single-center; lack of surgical correlation in all cases.

## 3 Diagnostic applications of FAPI PET/CT in AIDs

The diagnostic potential of FAPI PET/CT in AIDs lies in its ability to specifically target and visualize activated fibroblasts, which play a central role in the pathogenesis of chronic inflammation, tissue remodeling, and fibrosis. This section provides a detailed overview of its validated and emerging diagnostic applications across major AIDs categories.

### 3.1 Inflammatory arthritis

#### 3.1.1 Rheumatoid arthritis

Rheumatoid arthritis (RA) is a chronic systemic autoimmune disorder characterized by persistent polyarticular synovitis, leading to progressive cartilage destruction and bone erosion (6). Synovial fibroblasts play a pivotal role in RA pathogenesis, with single-cell transcriptomics identifying enriched fibroblast-specific gene signatures in refractory RA patients unresponsive to IL-6/CD20-targeted biologics (7). Consequently, molecular imaging of FAP using FAPI has been proposed as a potential diagnostic and predictive tool, enabling *in vivo* visualization of fibroblast-driven pathology across the RA disease continuum.

Remarkably, FAPI PET/CT exhibits predictive value in identifying individuals at high risk for developing clinical RA. In a pioneering study of 18 ACPA-positive individuals with clinically suspect arthralgia, [^68^Ga]Ga-FAPI-46 PET/CT revealed that synovial fibroblast activation occurs prior to clinical onset. The total lesion FAPI uptake (TLF) was a significant predictor of progression to clinical RA. This suggests that FAPI imaging can detect subclinical synovial pathology and stratify the risk of future disease development, potentially enabling pre-emptive intervention strategies (8).

In established disease, [^68^Ga]Ga-FAPI PET/CT holds particular diagnostic promise in seronegative RA, where conventional serological markers are negative. Imaging in such patients reveals intense, symmetrical tracer uptake across joints, clearly delineating synovial contours and providing detailed mapping consistent with an inflammatory arthritis pattern, thereby aiding diagnosis in clinically challenging cases (9).

Compared to X-ray, FAPI PET/CT provides superior functional assessment. A head-to-head comparison study confirmed that FAPI-derived parameters were significantly correlated with key clinical indicators including C-reactive protein (CRP), tender/swollen joint counts (TJC/SJC), DAS28, and SDAI. In stark contrast, no significant correlations were observed between any X-ray findings and these clinical activity parameters. This underscores FAPI PET/CT’s unique capacity to provide a quantitative functional assessment that anatomical imaging cannot achieve (10).

FAPI PET/CT demonstrates superior sensitivity and quantitative assessment of disease activity compared to FDG. A foundational prospective dual-tracer study provided direct comparative metrics: [^68^Ga]Ga-FAPI PET/CT detected 244 affected joints, all of which were FAPI-avid, while 6.1% (15/244) of these joints were missed by [^18^F]-FDG PET/CT (11). The maximum standardized uptake value (SUVmax) in the most affected joint was significantly higher with FAPI than with FDG. FAPI-derived composite scores, including the PET joint count and PET articular index, showed significant positive correlations with clinical disease activity variables and radiographic joint damage progression (11).

The validity of FAPI uptake as a quantitative imaging biomarker of disease activity is further substantiated by a study employing [^18^F]FAPI-42 PET/CT. It confirmed significantly elevated SUVmax in RA joints compared to controls. Patients with high disease activity, as measured by DAS28-CRP, demonstrated markedly increased FAPI uptake across multiple metrics, including SUVmax, target-to-background ratio (TBR), FAPI lesion volume (FLV), and TLF. Notably, strong positive correlations were observed between DAS28-CRP and both FLV and TLF (12).

#### 3.1.2 Spondyloarthritis

##### 3.1.2.1 Psoriatic arthritis

Psoriatic arthritis (PsA) is a complex inflammatory disorder affecting approximately 30% of individuals with psoriasis. It is characterized by heterogeneous clinical features, including cutaneous lesions, axial and peripheral joint involvement, enthesitis, and dactylitis (13). The molecular mechanisms underlying the progression from psoriasis to PsA remain incompletely elucidated. Currently, there is a lack of both reliable biomarkers and accurate imaging modalities for predicting this transition (14). Moreover, effective biomarkers or imaging techniques for the assessment of disease activity are also scarce.

FAPI PET/CT shows particular promise in identifying PsO patients at risk of progression to PsA. In a prospective cohort of 36 PsO patients with arthralgia, [^68^Ga]Ga-FAPI-04 PET/CT identified subclinical fibroblast activation in 29 patients (80.6%), involving 318 joints (7.9%) and 369 entheses (7.3%). SUVmax exhibited significant positive correlations with tender joint count and tender entheses count (15). In contrast, no significant correlation was observed with ultrasound findings. Furthermore, patients demonstrating significant synovio-entheseal FAPI uptake had a statistically higher risk of progressing to clinical PsA.

In established PsA, FAPI PET/CT provides comprehensive assessment of disease activity. A prospective dual-tracer study comparing [^68^Ga]Ga-FAPI-04 with [^18^F]NaF PET/CT revealed that FAPI PET joint count significantly correlated with multiple clinical measures including tender joint count, swollen joint count, and DAS28-CRP, whereas [^18^F]NaF showed no such correlations (16).

Additionally, [^68^Ga]Ga-FAPI PET/CT is valuable for assessing the severity of PsA. The [^68^Ga]Ga-FAPI-04 PET/CT revealed intense FAPI uptake in the spine, both feet, and the right hip of a patient with PsA who presented with a rash and back pain. This finding suggests that [^68^Ga]Ga-FAPI PET/CT could be a valuable tool for evaluating the condition of PsA (17).

##### 3.1.2.2 Axial spondyloarthritis

FAPI PET/CT demonstrates significant potential as a sensitive tool for the early diagnosis and systemic assessment of disease activity in axial spondyloarthritis/ankylosing spondylitis (axSpA/AS), effectively bridging the gap between structural and inflammatory evaluation.

A study utilizing [^18^F]AlF-NOTA-FAPI-04 PET/CT in 20 patients with active AS demonstrated its high diagnostic sensitivity, detecting positive tracer uptake in 46.7% of all assessed joints (1300/2820) and in 97.5% of sacroiliac joints (39/40). Notably, pathological uptake was identified in the sacroiliac joints of two patients without radiographic evidence of sacroiliitis, suggesting its utility for early diagnosis prior to the appearance of structural damage on conventional imaging. The derived quantitative metrics, including the positive joint count (PJC) and systemic joint SUV ratio (SUVR), showed significant positive correlations with most clinical assessments and laboratory parameters. Specifically, the sacroiliac joint SUVR correlated with serum CRP levels (18).

Similarly, a prospective study involving 22 axSpA patients compared with 12 healthy volunteers revealed significantly higher [^68^Ga]Ga-FAPI-46 uptake in multiple joints, particularly within the spine, in the patient cohort. The semi-quantitative FAPI uptake scores derived from these images demonstrated strong correlations with the Ankylosing Spondylitis Disease Activity Score (ASDAS), serum inflammatory markers such as CRP, and inflammation as visualized by MRI, further validating FAPI PET/CT as a quantitative biomarker of systemic disease activity (19).

A pivotal case report highlights the superior sensitivity of FAPI PET/CT over [^18^F]FDG PET/CT in diagnosing AS. In a patient with concurrent rectal adenocarcinoma and chronic low back pain, [^68^Ga]Ga-FAPI PET/CT revealed intense tracer uptake in the sacroiliac and costovertebral joints, indicative of active sacroiliitis and spondylitis. This finding stood in contrast to the negative [^18^F]FDG PET/CT scan. Subsequent clinical evaluation, including a positive HLA-B27 status, confirmed the diagnosis of AS (20). This case underscores the enhanced capability of FAPI PET/CT to detect active axial inflammation, even within complex clinical presentations complicated by concurrent malignancy.

##### 3.1.2.3 SAPHO syndrome

Synovitis, acne, pustulosis, hyperostosis, and osteitis (SAPHO) syndrome is a rare inflammatory disorder whose diagnosis is often challenging due to the nonspecific findings of conventional imaging modalities (21). By specifically targeting activated fibroblasts, FAPI PET/CT offers a novel imaging approach for the precise assessment of this condition.

A representative case illustrates the clinical utility of FAPI PET/CT. A 66-year-old woman presented with anterior chest wall and knee pain accompanied by multiple skin lesions. ^99m^Tc-MDP bone scintigraphy only indicated abnormal bone metabolism at the sternal angle, whereas [^68^Ga]Ga-DOTA-FAPI-04 PET/CT clearly demonstrated increased tracer uptake in the sternum and right knee joint. Subsequent ultrasound confirmed synovitis in the right knee, and sternal biopsy revealed no malignancy, leading to a final diagnosis of SAPHO syndrome (22). This case highlights FAPI imaging’s ability to provide a more comprehensive and intuitive visualization of active inflammatory sites.

Compared to ^99m^Tc-MDP bone scintigraphy, FAPI PET/CT shows higher sensitivity and enables simultaneous assessment of synovial inflammation. A prospective study comparing [^68^Ga]Ga-FAPI-04 PET/CT and ^99m^Tc-MDP bone scintigraphy in 19 SAPHO patients identified a total of 84 osteoarticular lesions. Bone scintigraphy detected 77 lesions (91.7%), while FAPI PET/CT detected 81 lesions (96.4%), demonstrating higher detection sensitivity. Additionally, FAPI PET/CT identified synovitis in the knee and hip joints in 5 patients, an assessment not achievable with bone scintigraphy alone (23).

Compared to ^18^F-FDG, [^68^Ga]Ga-FAPI PET/CT demonstrates higher lesion detection rates, stronger tracer uptake, and better clinical-imaging correlation. In a direct comparative study of 21 SAPHO patients, [^68^Ga]Ga-DOTA-FAPI-04 PET/CT detected a total of 38 involved sites, not only covering all 28 lesions identified by [^18^F]-FDG PET/CT but also revealing an additional 10 lesions. In skeletal involvement areas, FAPI tracer uptake and TBR were significantly higher than those of FDG. More importantly, the agreement between FAPI-positive lesions and current osteoarticular symptoms was substantial, whereas FDG showed only low-to-moderate agreement with clinical symptoms, indicating that FAPI imaging is superior in reflecting clinical disease activity (24).

Current evidence suggests that FAPI PET/CT may serve as a useful imaging tool to support early diagnosis, staging, and disease activity assessment in SAPHO syndrome, pending further validation.

### 3.2 Connective tissue disease

#### 3.2.1 IgG4-related disease

IgG4-Related Disease (IgG4-RD) is a fibroinflammatory disorder characterized by lymphoplasmacytic infiltration rich in IgG4-positive plasma cells and varying degrees of storiform fibrosis (25). Recent clinicopathologic classifications have distinguished between proliferative and fibrotic subtypes. Accurate assessment of fibroinflammatory activity and subtype differentiation is crucial for disease management (26). Recent advancements suggest that FAPI PET/CT imaging represents a promising non-invasive tool for evaluating IgG4-RD.

Compared to conventional imaging, FAPI PET/CT identifies a greater extent of organ involvement, with one prospective study reporting additional lesions in 50% of patients (27). TLF correlates significantly with serum IgG4 levels and the IgG4-RD Responder Index, establishing it as an objective imaging biomarker of disease burden (27).

In comparison to [^18^F]-FDG, [^68^Ga]Ga-FAPI exhibits higher sensitivity for fibrosis-predominant lesions, such as those affecting the pancreas, bile ducts, and salivary glands, detecting 13.2% more involved organs (28). Conversely, its low uptake in lymph nodes—which typically lack storiform fibrosis—confirms its specificity for fibrotic activity (28). This complementary role is further clarified through dual-tracer imaging, where [^18^F]-FDG corresponds histologically to IgG4+ plasma cell infiltration, while [^68^Ga]Ga-FAPI localizes to regions of activated FAP+ fibroblasts, enabling phenotypic distinction between inflammatory and fibrotic disease states (29).

Clinically, the ratio of [^68^Ga]Ga-FAPI to [^18^F]-FDG uptake (PET index) differentiates disease subtypes, with the proliferative form showing higher values than the fibrotic form (30). Importantly, a PET index ≥1.5 is associated with significantly shorter relapse-free survival, underscoring its prognostic utility (30). However, this threshold requires prospective validation in larger, independent cohorts before routine clinical application.

Together, this evidence indicates that [^68^Ga]Ga-FAPI PET/CT is not only a sensitive diagnostic tool but also an potential imaging biomarker capable of quantifying fibroinflammatory activity, differentiating disease subtypes, and predicting relapse risk.

#### 3.2.2 Systemic sclerosis

Systemic Sclerosis (SSc) is characterized by autoimmunity, vasculopathy, and progressive multi-organ fibrosis, presenting significant clinical challenges in the early detection and quantification of organ-specific fibrotic activity (31). FAPI PET/CT addresses this by targeting activated fibroblasts for direct visualization of fibrotic burden, while FDG PET/CT reflects inflammatory activity via GLUT1/GLUT3 in myeloid cells (32). FAPI PET/CT, a non-invasive molecular imaging technique, offering a novel approach for evaluating systemic fibrotic burden in SSc.

Whole-body FAPI PET/CT has been utilized to characterize the fibrotic landscape in SSc. A prospective study using [^18^F]AlF-NOTA-FAPI-04 in 31 patients revealed abnormal tracer uptake most frequently in the lungs (96.8%), followed by the heart (35.5%), kidneys, and skeletal muscles (12.9%) (33). Both whole-lung SUVmean and cardiac SUVmax were significantly higher in SSc patients than in controls. Strong correlations in tracer uptake were observed across organs (e.g., lung vs. kidney SUVmean: r=0.722), suggesting a systemic and coordinated fibrotic process (33).

Among these organs, pulmonary involvement is clinically paramount, as interstitial lung disease (ILD) accounts for 40% of SSc-related deaths within a decade (31). Bergmann et al. evaluated ^68^Ga-FAPI-04 PET/CT in 21 SSc-ILD patients, demonstrating significantly higher pulmonary tracer uptake versus controls, as reflected by SUVmean and SUVmax (34). Critically, elevated baseline FAPI uptake independently predicted subsequent ILD progression—even after adjusting for high-resolution computed tomography (HRCT) extent and forced vital capacity (FVC)—and correlated inversely with pulmonary function.

Beyond pulmonary involvement, cardiac fibrosis represents another critical yet underdiagnosed complication of SSc, with myocardial fibrosis (MF) serving as a key prognostic determinant. Despite its prognostic significance, current diagnostic modalities for MF lack sufficient specificity and sensitivity. A proof-of-concept study demonstrated that [^68^Ga]Ga-FAPI-04 PET/CT can visualize fibroblast activation in SSc-related MF (35). Among 14 SSc patients, those with MF showed significantly higher myocardial uptake, with elevated uptake associated with arrhythmias, increased NT-pro-BNP, and positive late gadolinium enhancement on cardiac MRI. Biopsy validation confirmed FAP+ fibroblasts and collagen deposition in high-uptake regions.

Extending these findings to pediatric populations, preliminary data highlight FAPI PET/CT’s potential in juvenile SSc (jSSc), where early detection of subclinical organ involvement is critical for improving long-term outcomes. Preliminary data in jSSc showed [^68^Ga]Ga-FAPI-46 PET/CT detected all known organ manifestations and revealed previously unknown cardiac/muscular involvement in 4 pediatric patients (36), supporting utility for subclinical multi-organ screening in early/pediatric disease.

Collectively, FAPI PET/CT may represent a useful quantitative imaging modality for evaluating systemic fibrotic burden in SSc, with potential to support early detection of multi-organ involvement and contribute to prognostic risk stratification.

#### 3.2.3 Idiopathic inflammatory myopathies

Idiopathic inflammatory myopathies (IIM) are rare AIDs characterized by skeletal muscle weakness, muscular atrophy, and significant physical disability accompanied by extra-muscular complications including arthritis, cardiac issues, microvascular problems, dermatological manifestations, and pulmonary involvement (37). The leading causes of mortality in this population include malignancies, cardiovascular diseases, and pulmonary complications (37). Evidence of ongoing tissue remodeling in IIM-ILD remains sparse, and predicting disease progression continues to be challenging.

By targeting activated mesenchymal stromal cells within muscle, FAPI PET/CT enables an objective and quantitative evaluation of myositis activity. In a study of 26 IIM patients, whole-body [^18^F]FAPI-42 PET/CT showed significantly elevated global parameters compared to controls, including FAPI-avid muscle ratio and muscle TBR. These parameters correlated strongly with MRI edema scores, muscle strength, and serum creatine kinase levels, establishing FAPI as a sensitive biomarker for muscular inflammatory and fibrotic burden (38).

In IIM-ILD, FAPI PET/CT demonstrates high sensitivity for detecting activated pulmonary fibroblasts and has been associated with disease progression in preliminary studies. A prospective analysis of 32 patients revealed that whole-lung FAPI uptake parameters (wlTBRmax and wlTBRmean) were significantly higher in IIM-ILD patients compared to both non-ILD IIM patients and those with ILD of other etiologies (39). Patients with progressive ILD exhibited significantly elevated baseline wlTBRmax and wlTBRmean compared to those with stable disease. wlTBRmean correlated positively with the extent of disease on high-resolution CT and the severity of respiratory symptoms, while inversely correlating with FVC and diffusing capacity of the lungs for carbon monoxide (DLCO) (39). A separate longitudinal study of 14 IIM-ILD patients further confirmed that individuals meeting the INBUILD progression criteria within two years had significantly higher baseline pulmonary FAPI uptake (40). In MDA5-positive dermatomyositis, [^68^Ga]Ga-FAPI-04 PET/CT showed more pronounced pulmonary uptake than [^18^F]FDG PET/CT, suggesting its utility in assessing fibrotic activity (41).

The modality also facilitates simultaneous evaluation of myositis and occult malignancy. In a case of dermatomyositis, [^68^Ga]Ga-FAPI PET/CT detected diffuse muscular uptake alongside a focal nasopharyngeal lesion later confirmed as carcinoma (42), demonstrating its dual diagnostic capability.

In summary, FAPI PET/CT may serve as a useful tool for assessing muscular disease activity, screening for associated malignancies, and evaluating the risk of ILD progression in IIM.

#### 3.2.4 Lupus nephritis in systemic lupus erythematosus

Systemic lupus erythematosus (SLE) is a prototypical autoimmune connective tissue disease characterized by dysregulated immune activation, which drives the production of diverse autoantibodies targeting multiple tissues—with the kidneys exhibiting particular susceptibility. Notably, approximately 50% of patients with lupus nephritis (LN) progress to end-stage renal disease (ESRD). Emerging evidence supports FAPI PET/CT as a promising noninvasive modality for assessing renal fibrosis in LN (43).

In a prospective study of 29 biopsy-proven LN patients, renal [^68^Ga]Ga-FAPI-04 uptake was significantly higher than in 26 healthy controls (44). TBR correlated strongly with serum creatinine levels and renal biopsy chronicity index, particularly in patients with a chronicity index >4 who exhibited markedly elevated TBR (44). Histopathological analysis confirmed that FAPI uptake correlated with tubulointerstitial fibrosis extent on Masson trichrome staining, with FAP expression localized predominantly in renal tubular epithelial cells and inversely associated with E-cadherin expression, suggesting epithelial–mesenchymal transition (44). Mechanistically, transcriptomic analysis of LN renal tissue revealed significant enrichment of type I interferon signaling pathways, which correlated with FAP mRNA expression. *In vitro* stimulation of renal tubular epithelial cells with interferon-α resulted in significant upregulation of both FAP mRNA (≥2-fold increase) and protein expression, establishing a molecular link between SLE-related immune dysregulation and fibrogenesis (44).

### 3.3 Large vessel vasculitis

Large vessel vasculitis (LVV) is a chronic, immune-mediated inflammatory disorder characterized by non-specific granulomatous inflammation, predominantly affecting young Asian women (46). The disease is distinguished by its early onset, protracted course, and elevated mortality rate. As the disease progresses, fibroblasts may significantly contribute to the pathogenesis of vasculitis by influencing the extracellular matrix (ECM), secreting proinflammatory molecules and differentiating into myofibroblasts, which play a role in vascular remodeling (46).

Emerging evidence has highlighted the promising role of FAPI PET/CT in the evaluation of systemic vasculitis, particularly large vessel vasculitis (LVV). In a retrospective study of 30 patients with systemic vasculitis (17 LVV, 10 ANCA-associated vasculitis, 2 Behçet’s disease, 1 polyarteritis nodosa) undergoing dual-tracer imaging, [^18^F]FAPI-42 PET/CT demonstrated superior diagnostic performance compared to [^18^F]FDG PET/CT. While both modalities showed high positivity rates, [^18^F]FAPI detected significantly more lesions (161/168 vs. 145/168) and revealed more extensive vascular involvement in 60% (18/30) of patients. Quantitative analysis showed comparable SUVmax values, but significantly higher TBR for [^18^F]FAPI, indicating superior lesion contrast (47). Notably, FAPI uptake shows a moderate correlation with systemic inflammatory markers such as ESR and CRP, supporting its relevance to disease activity (47).

Notably, FAPI imaging provides unique insights into vascular wall remodeling beyond acute inflammation. In a study of LVV patients including both active (n=3) and remitted cases (n=5), FAPI uptake remained visually identifiable in 80% (4/5) of clinically remitted patients despite nearly negative MRI inflammatory scores (48). This persistent uptake suggests ongoing fibroblast activation during the fibrotic remodeling phase. The clinical utility of FAPI PET/CT for therapeutic monitoring is demonstrated by follow-up data from 6 patients showing decreased SUVmax, TBR values, and number of detected lesions paralleling clinical remission (48). Furthermore, in a diagnostically challenging case of active Takayasu arteritis with undetectable blood pressure in bilateral arms and elevated inflammatory markers, [^68^Ga]Ga-FAPI-04 PET/CT demonstrated significant uptake in the arterial walls while initial [^18^F]FDG PET/CT showed no abnormal uptake, highlighting FAPI’s potential superior sensitivity in detecting active vascular inflammation (49).

Collectively, these findings suggest that FAPI PET/CT may serve as a sensitive diagnostic tool with high lesion contrast and as a potential biomarker for detecting persistent fibroblast activity during vascular remodeling. This could have implications for disease staging and treatment monitoring in systemic vasculitis, although further validation is required.

### 3.4 Crohn’s disease

Crohn’s disease (CD) represents a chronic, immune-mediated inflammatory bowel disorder characterized by transmural inflammation and progressive intestinal fibrosis (50). Accurate differentiation between inflammatory and fibrotic components is critical for therapeutic decision-making, yet remains challenging with conventional imaging. FAP+ fibroblasts are crucial in the fibrogenesis that leads to excessive extracellular matrix buildup in CD (51).

Animal models reveal distinct dynamics of [^18^F]FAPI uptake during fibrogenesis, peaking in early stages (3–6 weeks) and declining later despite persistent fibrosis, reflecting selective visualization of activated rather than quiescent fibroblasts. These findings provide mechanistic context for interpreting clinical FAPI-PET results in CD (52).

[^68^Ga]Ga-FAPI-04 PET/CT demonstrates high sensitivity (93.3%) in identifying endoscopically active lesions, outperforming CT enterography (CTE) (86.7%) (53). The TBR correlates strongly with CTE score and the Simple Endoscopic Score for CD, supporting its role in activity assessment (53). Furthermore, whole-body FAPI uptake scores show significant correlations with fecal calprotectin, CRP, and the CD Activity Index, confirming its utility as a non-invasive biomarker of disease activity (53).

The ability of using [^68^Ga]Ga-FAPI-04 to distinguish fibrotic from inflammatory strictures is well established. Fibrotic bowel segments exhibit significantly higher SUVmax than non-fibrotic segments, with severe fibrosis showing greater uptake than mild-to-moderate fibrosis (54, 55). Importantly, purely inflammatory segments demonstrate lower uptake than those with mixed inflammation and fibrosis (55).

FAPI-PET demonstrates superior diagnostic performance relative to [^18^F]FDG PET/CT. A prospective head-to-head study reported higher sensitivity and specificity for FAPI-PET, along with a significantly greater AUC (56). Meta-analytic data further support its advantage, showing that FAPI-PET detects fibrotic lesions with 99% overall sensitivity and approximately twice the diagnostic efficacy of FDG-PET. Pooled analyses reveal a marked SUVmax difference between fibrotic and non-fibrotic segments and a strong correlation with histological fibrosis grading (57).

Current evidence indicates that FAPI-PET may represent a useful functional imaging tool in CD, demonstrating high sensitivity for active inflammation and potential utility in discriminating between fibrotic and inflammatory pathology, with favorable performance compared to FDG-PET in preliminary studies.

## 4 FAPI PET/CT in guiding treatment and monitoring of AIDs

FAPI PET/CT is evolving from a diagnostic tool toward potential applications in AIDs management. By quantifying pathogenic fibroblast activation *in vivo*, it provides biological insights that may inform patient stratification, disease monitoring, and prognostic assessment. However, while observational studies have shown correlations between FAPI uptake and clinical outcomes or post-therapy changes, its role in actively guiding treatment remains investigational. Current evidence does not yet demonstrate improved patient outcomes, and prospective trials comparing FAPI-guided strategies against standard care are lacking.

### 4.1 Predicting and monitoring treatment response in RA and SpA

Baseline FAPI PET/CT parameters serve as potent predictive biomarkers for therapeutic response. In RA patients, significantly higher baseline levels of PET joint count (PJC_FAPI_), total synovitis uptake (TSU_FAPI_), and metabolic synovitis volume (MSV_FAPI_) were observed in individuals who subsequently achieved an early clinical response at 3-month follow-up compared to non-responders (58). This predictive capacity surpasses that of conventional radiography, which fails to differentiate future responders (10). Following treatment initiation, serial FAPI imaging enables objective monitoring. A significant decrease in synovial and entheseal FAPI uptake has been documented following successful cytokine inhibition (e.g., with TNF or IL-17A antagonists), aligning temporally with clinical resolution (59). This provides a molecular-level confirmation of target engagement and treatment efficacy.

These preliminary observations suggest that baseline FAPI PET parameters may hold predictive value, but the proposed thresholds require validation in larger, independent cohorts. Prospective studies are needed to determine whether treatment adjustments based on FAPI findings translate into improved clinical outcomes.

### 4.2 Guiding therapeutic strategy in IgG4-RD

FAPI PET/CT is instrumental in personalizing treatment by differentiating disease subtypes with distinct therapeutic implications. It effectively distinguishes between inflammatory-dominant lesions (high [^18^F]FDG, low FAPI) and fibrosis-dominant lesions (high FAPI). This stratification is clinically critical: the former may optimally respond to immunosuppressive therapy, while the latter, often refractory to anti-inflammatory agents alone, might necessitate adjunctive antifibrotic strategies (30).

### 4.3 Risk stratification and treatment monitoring in SSc and IIM

In SSc-ILD, FAPI PET/CT provides valuable prognostic information and can inform treatment intensity. Baseline pulmonary FAPI uptake independently predicts future disease progression and correlates with subsequent decline in lung function, regardless of baseline CT findings or pulmonary function test results (34). This capability enables early identification of high-risk patients who may benefit from closer monitoring or timely initiation of antifibrotic therapy. Furthermore, FAPI PET/CT serves as a sensitive tool for monitoring treatment response. Studies have demonstrated that a reduction in pulmonary [^68^Ga]Ga-FAPI-04 uptake during treatment with nintedanib correlates with subsequent improvements in lung function, establishing it as an early imaging biomarker of therapeutic efficacy (34). The prognostic utility of FAPI PET/CT extends to IIM-ILD. Baseline quantitative pulmonary uptake parameters (wlTBRmax, wlTBRmean) are significantly elevated in patients who subsequently experience progressive ILD, as defined by stringent criteria like the INBUILD progression criteria (42). While baseline pulmonary FAPI uptake appears to offer a more responsive measure of fibrotic activity and treatment response, prospective management-impact studies are needed to determine whether FAPI-guided antifibrotic therapy improves long-term outcomes.

Beyond the lungs, FAPI PET/CT also shows promise in assessing cardiac involvement in SSc. In SSc-MF, [^68^Ga]Ga-FAPI-04 uptake correlates moderately with extracellular volume measured by cardiac MRI. Sequential PET imaging has shown a significant 42% ± 8% reduction in tracer uptake in patients with clinical improvement, while conventional cardiac MRI parameters remained stable (35).

### 4.4 Predicting treatment response in LN

FAPI PET/CT shows potential for predicting treatment response in LN. In a study of 20 LN patients, baseline renal [^18^F]FAPI SUVmax correlated inversely with eGFR and positively with ESR, while [^18^F]FDG uptake showed no significant correlation (45). After six months of therapy, 50% achieved complete renal response (CR), 25% partial response, and 25% no response. CR patients had significantly lower baseline FAPI SUVmax and TBR compared to non-responders. Visual FAPI assessment outperformed FDG in predicting CR, with higher accuracy (85% vs. 70%), specificity (90% vs. 50%), and positive predictive value (89% vs. 64%). Multivariate analysis identified negative baseline FAPI uptake as the sole independent predictor of CR (45).

Renal FAPI uptake correlates with chronicity and treatment non-response, yet prospective trials are required to validate its role in guiding immunosuppression versus antifibrotic therapy. Prediction of long-term renal function preservation remains unestablished.

### 4.5 Informing clinical decisions in CD

By precisely distinguishing surgically-indicated fibrotic strictures from inflammation-dominant strictures amenable to medical therapy, FAPI PET/CT directly guides critical treatment decisions in CD management. A recent study suggests that strictures with high SUVmax uptake (≥3.5) are indicative of fibrosis-predominant pathology and warrant consideration for surgical intervention. Conversely, strictures with low SUVmax uptake (<3.5) suggest inflammation-driven pathology, for which intensified medical therapy is the preferred initial approach (54). However, it must be emphasized that the SUVmax threshold of 3.5 was derived specifically from [^68^Ga]Ga-FAPI-04 in single-center cohorts. Its generalizability may be limited by factors such as tracer heterogeneity, protocol variability, and patient selection. Accordingly, its clinical applicability requires multicenter validation and correlation with histopathological findings.

## 5 Key limitations of current evidence and challenges to clinical implementation

The evidence summarized herein indicates that FAPI PET/CT holds considerable promise for visualizing fibroblast activation in AIDs, offering new insights into disease pathophysiology and tissue remodeling. However, its transition from research to routine clinical application faces significant hurdles related to methodological standardization, diagnostic specificity, and clinical validation. Addressing these challenges systematically is essential for realizing its full potential.

### 5.1 Heterogeneity in tracers and protocols

Tracer heterogeneity represents a fundamental and often underappreciated limitation in the current FAPI PET literature. Variability arises at multiple levels: the radionuclide (^68^Ga vs. ^18^F), which affects half-life, image resolution, and scan logistics; the chelator or linker scaffold (DOTA, NOTA, quinoline-based), which modulates pharmacokinetics and off-target binding; and the inhibitor variant (FAPI-04, -46, -42), each with distinct biodistribution and target-to-background profiles. As a result, quantitative metrics such as SUVmax, TBR, and TLF are not interchangeable across tracers or protocols. Thresholds established with one tracer cannot be extrapolated to others without dedicated, tracer-specific validation. **Table 2** summarizes the key characteristics of commonly used FAPI tracers in AIDs, including radionuclide properties, physiologic uptake, pitfalls, and representative studies.

**Table 2:** Characteristics of FAPI tracers commonly reported in AIDs.

Tracer (common name)	Radionuclide (half-life)	Typical administered activity	Key physiologic & non-target uptake	Notable advantages/challenges in AIDs	Exemplary studies in this review
[^68^Ga]Ga-DOTA-FAPI-04	^68^Ga (~68 min)	1.5–2.5 MBq/kg	Pancreas, uterus, salivary glands, healing bone fractures, surgical scars. Renal excretion.	Most extensively studied; wide availability via ^68^Ge/^68^Ga generators. High pancreatic uptake may obscure pancreatitis.	RA, PsA, SAPHO, IgG4-RD, CD
[^18^F]AlF-NOTA-FAPI-04	^18^F (~110 min)	2.0–3.0 MBq/kg	Similar to [^68^Ga]Ga-FAPI-04.	Better image resolution due to ^18^F; allows delayed imaging; requires cyclotron.	axSpA/AS, SSc, IgG4-RD
[^68^Ga]Ga-FAPI-46	^68^Ga (~68 min)	1.5–2.5 MBq/kg	Reportedly lower hepatobiliary & intestinal uptake vs. FAPI-04.	Potentially improved tumor-to-background ratio; less experience in AIDs.	RA (at-risk), axSpA, jSSc
[^18^F]F-FAPI-42	^18^F (~110 min)	2.0–3.0 MBq/kg	Data still emerging; likely similar to other FAPI tracers.	^18^F advantages; used in newer quantitative studies.	RA, Systemic Vasculitis

### 5.2 Diagnostic specificity and confounders

FAPI PET targets activated fibroblasts, a process central to fibrosis but also present in numerous other conditions including tissue repair, degenerative changes, infection, and malignancy. Thus, FAPI uptake reflects “process specificity” rather than “disease specificity.” Most studies reviewed herein did not systematically address these confounders. To improve diagnostic accuracy, FAPI PET findings must be interpreted in conjunction with clinical context, physical examination, and correlative anatomical imaging (CT/MRI). Histopathological confirmation remains essential in uncertain or suspicious cases.

### 5.3 Key evidence gaps for clinical translation

Despite encouraging preliminary data, establishing FAPI PET/CT as a routine management tool for AIDs still requires bridging several evidence gaps. First, the reproducibility of its quantitative metrics and their correlation with histopathological “gold standards” must be validated in prospective, multicenter cohorts. Second, beyond correlations with surrogate endpoints, there is an urgent need for management impact studies to prospectively assess whether adjusting treatment strategies based on FAPI PET results ultimately improves patient-centered key clinical endpoints (e.g., preservation of organ function, quality of life, progression-free survival). Furthermore, a deeper understanding of the dynamic relationships among FAPI signals, specific fibroblast functional subtypes, the local immune microenvironment, and final clinical outcomes (reversible inflammation versus irreversible fibrosis) is essential. Finally, comprehensive health economic analyses are crucial for clarifying the cost-effectiveness advantages of FAPI PET/CT compared to existing imaging modalities (e.g., ultrasound, MRI) in specific clinical scenarios. This will provide key evidence for its rational clinical integration and resource allocation.

## 6 Key limitations at a glance

- Tracer heterogeneity
- Physiologic uptake
- Scanner/protocol variability
- Partial volume effects
- Motion artifacts (especially thoracic)
- Renal excretion confounders
- Lack of test-retest reproducibility data

## 7 Conclusion and future perspectives

In summary, FAPI PET/CT emerges as a promising functional imaging modality that offers new insights into the visualization and assessment of fibroblast-driven pathological processes in AIDs. To realize its clinical potential, however, methodical and collaborative efforts are required to address existing methodological variability, substantiate its diagnostic utility, and validate its impact on patient outcomes. A rigorous translational pathway will be essential to determine the appropriate role of FAPI PET/CT within a precision medicine framework for AIDs.
